# WIfI classification: the Society for Vascular Surgery lower extremity threatened limb classification system, a literature review

**DOI:** 10.1590/1677-5449.190070

**Published:** 2020-05-08

**Authors:** Lorena de Oliveira Cerqueira, Eliud Garcia Duarte, André Luis de Souza Barros, José Roberto Cerqueira, Walter Júnior Boim de Araújo

**Affiliations:** 1 Universidade Vila Velha – UVV, Cirurgia Vascular, Vila Velha, ES, Brasil.; 2 Centro de Atuação Precoce em Úlceras Vasculares e Complicações do Pé Diabético – PROPÉ, Departamento de Cirurgia Vascular, Vila Velha, ES, Brasil.; 3 Hospital Estadual de Urgência e Emergência do Espírito Santo – HEUE, Departamento de Cirurgia Vascular, Vila Velha, ES, Brasil.; 4 Hospital Santa Mônica de Vila Velha, Serviço de Cirurgia Vascular e Endovascular, Vila Velha, ES, Brasil.; 5 Universidade Federal do Paraná – UFPR, Hospital de Clínicas, Serviço de Angiorradiologia e Cirurgia Endovascular, Curitiba, PR, Brasil.; 6 Hospital Angelina Caron, Departamento de Residência Médica em Cirurgia Vascular e Endovascular, Curitiba, PR, Brasil.

**Keywords:** diabetic foot, foot ulcer, ischemia, infection, amputation, mortality

## Abstract

The Society for Vascular Surgery has proposed a new classification system for the threatened lower limb, based on the three main factors that have an impact on limb amputation risk: Wound (W), Ischemia (I) and foot Infection (“fI”) - the WIfI classification. The system also covers diabetic patients, previously excluded from the concept of critical limb ischemia because of their complex clinical condition. The classification’s purpose is to provide accurate and early risk stratification for patients with threatened lower limbs; assisting with clinical management, enabling comparison of alternative therapies; and predicting risk of amputation at 1 year and the need for limb revascularization. The objective of this study is to collect together the main points about the WIfI classification that have been discussed in the scientific literature. Most of the studies conducted for validation of this classification system prove its association with factors related to limb salvage, such as amputation rates, amputation-free survival, prediction of reintervention, amputation, and stenosis (RAS) events, and wound healing.

## INTRODUCTION

The first concept of critical limb ischemia (CLI) emerged in 1982 and since then the world has gone through countless changes, including advances in medicine and changes to the profile of patients. Originally, CLI was defined as systolic blood pressure in the ankle (ankle pressure, AP) < 40 mmHg, with pain at rest and AP < 60 mmHg with tissue necrosis.^1^^,^^2^ Diabetic patients were excluded from this concept, as explained at the end of the document published in 1982:

It was generally agreed that diabetic patients who have a varied clinical picture of neuropathy, ischemia and sepsis make definition even more difficult and it is desirable that these patients be excluded…or should be clearly defined as a separate category to allow the analysis of the results in the nondiabetic patients^3^ (p. S2-3).

The objective of the initial concept was therefore to define a group of patients without diabetes who had a threatened lower limb (TLL) because of chronic ischemia which would inevitably be lost without revascularization.^3^

In 2016, the World Health Organization (WHO) published a report to mark World Health Day in which it defined diabetes as a global epidemic. The document stated that the number of adults living with diabetes had quadrupled since 1980, reaching 422 million in 2014 and reflecting increases in the risk factors associated with it, such as overweight and obesity.^4^ The higher number of diabetic patients brought with it an increase in the incidence of diabetic foot ulcers (DFU) and peripheral arterial disease.^5^ In other words, the profile of the most common patient, which for 40 years had been a male, non-diabetic, smoker with atherosclerosis, had changed. In addition to these changes, there has been considerable modernization of revascularization techniques and other treatment approaches, which are available to vascular surgeons as alternative treatment options and must be compared and chosen individually.^2^

## PREVIOUS SYSTEMS AND THE NEED FOR A NEW CLASSIFICATION

It has already been confirmed that in the overall complexity of a TLL (regardless of whether the patient has diabetes), perfusion is just one determinant of the result, while characteristics of the wound and presence and severity of infection are also factors that have a major impact on the risk of limb amputation.^6^^,^^7^

These findings have revealed certain problems with the concept of CLI and the current classifications of TLLs. The first problem is that the foundation and natural history underlying the concept of CLI were based on determination of a critical value below which perfusion of the limb would be inadequate and so, in the absence of revascularization, the limb would inevitably be amputated. However, studies have demonstrated that the limbs of patients with CLI can be preserved even if they do not undergo revascularization.^1^^,^^2^ One example is a Swedish study by Elgzyri et al.^8^ that assessed 602 patients with DFU and systolic blood pressure in the toe (toe pressure, TP) < 45 mmHg or AP < 80 mmHg who were not revascularized. The study reported that 50% had favorable outcomes with only treatment of wounds or minor amputation; 17% improved after major amputation; and 33% died with intact limbs, but with unhealed wounds.

The second problem is that the original concept of CLI does not include diabetics.

The third problem is that the previously-existing classification systems for threatened limbs are limited, because they do not generally cover all three pillars (wound, ischemia, and infection) of the extremity at risk of amputation or do not differentiate ulcer from gangrene and so fail to encompass the entire heterogeneous nature of the causes and clinical presentations of TLLs. For example, the Rutherford et al.^9^ and Fontaine et al.^10^ classifications (Table 1) are primarily based on the degree of ischemia and the Wagner^11^ classification (Table 2), which is still widely used for wounds of the diabetic foot, is not much help for differentiating between the causes of ischemic and infectious gangrene.^12^ Meanwhile, the 2007 Inter-Society Consensus for the Management of Peripheral Arterial Disease (TASC II) stated that 20% of patients with chronic CLI would die in the first year of clinical presentation of the disease; that 10% of patients amputated at levels below the knee would die during the perioperative period; and that 2 years after surgery 30% would be dead.^13^ There was therefore an urgent need to create a staging system that could provide precise and early risk stratification for patients in relation to the natural history of the disease, encompassing the three most important factors that influence risk of limb amputation and clinical management. There was also a need for a tool that would enable a professional to make a significant comparison between the different treatments available and would help with clinical decision making.^1^^,^^14^

**Table 1:** Fontaine et al.^10^ and Rutherford et al.^9^ peripheral arterial disease classifications.

**Fontaine et al.** **^10^**		**Rutherford et al.** **^9^**		
**Stage**	**Clinical presentation**	**Grade**	**Category**	**Clinical presentation**
I	Asymptomatic	0	0	Asymptomatic
IIa	Mild claudication	I	1	Mild claudication
IIb	Moderate to severe claudication	I	2	Moderate claudication
III	Ischemic pain at rest	I	3	Severe claudication
IV	Gangrene or ulceration	II	4	Ischemic pain at rest
		III	5	Minor tissue lesion
		III	6	Major tissue lesion

**Table 2:** Wagner et al.^11^ classification of the diabetic foot.

**Grade**	**Characteristics**
0	Foot at risk, without evident ulcer, with thick calluses, prominent metatarsian heads, clawed toes, or other bone deformities
1	Uninfected superficial ulcer
2	Deep ulcer, bone not involved
3	Deep ulcer with formation of abscess or bone involvement
4	Localized gangrene in part of the foot
5	Extensive gangrene of the complete foot

In response to this, the Society for Vascular Surgery (SVS) developed a new classification system that dispenses with the term CLI and is based on the characteristics of the wound (W), on the degree of ischemia (I), and on the presence and severity of foot infection (fI): the SVS Wound, Ischemia and foot Infection classification, or WIfI classification.^1^^,^^2^

The objective of this study is to collect the most important points related to the WIfI classification that have been covered in scientific publications, since the original SVS article was published in 2014.^1^

## THE SVS WIFI CLASSIFICATION

The SVS classification system was developed in 2013 and proposed in an SVS publication in 2014 and covers the three most important parameters that put a limb at risk of amputation: wound, ischemia, and foot infection.^1^ The SVS WIfI classification attributes a 4-grade scale to each letter or parameter, running from 0 to 3, where 0 represents absent, 1 mild, 2 moderate, and 3 severe (Table 3). After grading, the scores attributed to each letter are combined and analyzed using two tables: one gives an estimation of the risk of amputation at 1 year and the other an estimation of the need for/benefit of revascularization (Tables 4 and 5). Based on the results, the limb is classified for risk of amputation and need of revascularization at clinical stages 1, 2, 3, or 4: very low, low, moderate, and high risk of amputation or benefit from revascularization, respectively (Tables 4 and 5). Stage 5 is reserved for feet that cannot be saved, even with revascularization. The stages were developed by a panel of specialists who used the Delphi method to arrive at a consensus categorization for each of the 64 possible combinations in the classification table.^1^

**Table 3:** The WIfI classification for threatened lower limbs: assessment of risk of amputation.^1^

**Component**	**Grade**	**Description**		
Wound (**W)**	**0**	No ulcer or gangrene (ischemic pain at rest)		
	**1**	Small or superficial ulcer on leg or foot, without gangrene (SDA or SC)		
	**2**	Deep ulcer with exposed bone, joint, or tendon ± gangrene limited to digits (MAD or standard TMA ± SC)		
	**3**	Deep, extensive ulcer involving forefoot and/or midfoot ± calcaneal involvement ± extensive gangrene (CR of the foot or nontraditional TMA)		
Ischemia (**I)**		**ABI**	**SBP of the ankle**	**TP, TcPO_2_**
	**0**	≥ 0.80	> 100 mmHg	≥ 60 mmHg
	**1**	0.6-0.79	70-100 mmHg	40-59 mmHg
	**2**	0.4-0.59	50-70 mmHg	30-39 mmHg
	**3**	≤ 0.39	< 50 mmHg	< 30 mmHg
foot Infection (**fI**)	**0**	Uninfected		
	**1**	Mild local infection, involving only the skin and subcutaneous tissue, erythema > 0.5 to ≤ 2 cm		
	**2**	Moderate local infection, with erythema > 2 cm or involving deeper structures		
	**3**	Severe local infection with signs of SIRS		

WIfI = Wound, Ischemia, and foot Infection; SDA = simple digital amputation; SC = skin coverage; MDA = multiple digital amputations; TMA = transmetatarsal amputation; CR = complex reconstruction; ABI = ankle-brachial index; SBP = systolic blood pressure; TP = toe pressure (SBP of toe); TCPO_2_ = transcutaneous oxygen pressure; SIRS = systemic inflammatory response syndrome.

**Table 4:** Estimation of risk of amputation at 1 year, according to WIfI classification clinical stages, as proposed by expert panel.^1^

	**Ischemia 0**				**Ischemia 1**				**Ischemia 2**				**Ischemia 3**			
Wound 0	VL	VL	L	M	VL	L	M	H	L	L	M	H	L	M	M	H
Wound 1	VL	VL	L	M	VL	L	M	H	L	M	H	H	M	M	H	H
Wound 2	L	L	M	H	M	M	H	H	M	H	H	H	H	H	H	H
Wound 3	M	M	H	H	H	H	H	H	H	H	H	H	H	H	H	H
	fI 0	fI 1	fI 2	fI 3	fI 0	fI 1	fI 2	fI 3	fI 0	fI 1	fI 2	fI 3	fI 0	fI 1	fI 2	fI 3

VL = very low; L = low; M = moderate; H = high; fI = foot infection; WIfI = Wound, Ischemia, and foot Infection.

**Table 5:** Estimate of need for/benefit of revascularization, according to the WIfI classification clinical stages, as proposed by expert panel. Infection must be controlled.^1^

	**Ischemia 0**				**Ischemia 1**				**Ischemia 2**				**Ischemia 3**			
Wound 0	VL	VL	VL	VL	VL	L	L	M	L	L	M	M	M	H	H	H
Wound 1	VL	VL	VL	VL	L	M	M	M	M	H	H	H	H	H	H	H
Wound 2	VL	VL	VL	VL	M	M	H	H	H	H	H	H	H	H	H	H
Wound 3	VL	VL	VL	VL	M	M	M	H	H	H	H	H	H	H	H	H
	fI 0	fI 1	fI 2	fI 3	fI 0	fI 1	fI 2	fI 3	fI 0	fI 1	fI 2	fI 3	fI 0	fI 1	fI 2	fI 3

VL = very low; L = low; M = moderate; H = high; fI = foot infection; WIfI = Wound, Ischemia, and foot Infection.

The system was created to precisely define the burden of the disease, but not with the intention of dictating the treatment method, since therapeutic modalities are continually evolving. It is therefore intended to be analogous for TLL to what the Tumor, Nodule, Metastasis (TNM) classification is for cancer, aiding in clinical management and enabling comparisons between similar groups of patients and alternative treatments.^1^^,^^2^^,^^15^

The target population for this system includes any patient with ischemic pain at rest, typically in the forefoot; with confirmatory objective hemodynamic studies (ankle-brachial index [ABI] < 0.40, AP < 50 mmHg, TP < 30 mmHg and/or transcutaneous O_2_ pressure [TcPO_2_] < 20 mmHg); DFU; and with ulceration of the foot or unhealed lower limb wound (lower limb) with duration ≥ 2 weeks and/or gangrene of lower limb or foot. The following conditions are excluded: patients with pure venous ulcers; wounds related to non-atherosclerotic conditions (for example: vasculitis, collagen vascular disease, Buerger’s disease, radiation); acute limb ischemia; acute trash foot or ischemia due to emboli; acute trauma/mutilated extremity.^1^

### W: wound/clinical category

In the SVS WIfI classification, the wound is classified according to its size, depth, severity, and (in contrast with previous classifications) the complexity of the procedure that is most likely needed for it to heal. Additionally, gangrene is included and stratified by extent of involvement (Table 3).^1^^,^^2^

### I: ischemia

The degree of ischemia can be measured using ABI, which, if ≥ 0.80, is classified as grade 0. If ABI is incompressible (> 1.3), TP or TcPO_2_ should be measured. Measurement of TP is obligatory in all patients with diabetes, because ABI could be falsely elevated because of calcifications. If ABI and TP result in different grades, TP will be the principal determinant of the degree of ischemia (Table 3).^1^^,^^2^

### fI: foot infection

The WIfI classifies presence and severity of infection, taking into consideration the earlier Perfusion, Extent, Depth, Infection and Sensation (PEDIS) and Infectious Diseases Society of America (IDSA) diabetic foot classification systems. The patient already shows systemic signs of infection if fI is defined as grade 3 or severe (Table 3).^1^^,^^2^

To exemplify: a 56-year-old man, without diabetes, has ischemic pain at rest, but no wounds. His ABI is 0.36 and there are no signs of local or systemic infection. He could be classified as wound 0, ischemia 3, and foot infection 0, or WIfI 030. His clinical stage would be 2 (low risk of amputation at 1 year) and revascularization benefit would be moderate (Tables 4 and 5).

## VALIDATION OF THE SYSTEM: CORRELATIONS WITH EXPECTED AND UNEXPECTED OUTCOMES

Since the original publication by Mills et al.,^1^ several important studies have been published that aimed to test and validate the SVS WIfI classification system (Table 6). Below, we present the most important studies published to date that discuss validation of the system and describe its advantages, limitations, and challenges for the future. We have divided the outcomes studied didactically. However, in the majority of the studies, certain outcomes have strong correlations.

**Table 6:** Principal studies on which this article is based, published to test and validate the SVS WIfI classification, and their Conclusions.

**Study**	**Nº of threatened limbs**	**Conclusions and important topics**
Cull et al.^15^	151	- The higher the WIfI clinical stage, the greater the risk of amputation at 1 year and the worse the healing of wounds.
		- Diabetes was independently associated with a higher risk of amputation at 1 year.
Zhan et al.^12^	201	- As WIfI clinical stage increases, the risk of major amputation increases, 1-year AFS reduces, and time taken for healing is prolonged.
		- Revascularization reduced the time taken for wounds to heal in patients at stage 3.
Darling et al.^16^	551	- The WIfI classification was able to predict risk of amputation at 1 year and rate of wound healing in patients with CLI who had already undergone below-the-knee endovascular treatment.
Causey et al.^17^	160	- This study recommends use of the WIfI classification and the Project of Ex-Vivo vein graft Engineering via Transfection III (PIII) as supplementary tools for management of CLTI.
		- The WIfI classification had a good correlation with length of hospital stay and with severe adverse limb events, such as amputation, over the medium term. - Rates of revascularization increase with WIfI stage. It was suggested that the classification is capable of predicting patient benefit from revascularization.
Beropoulis et al.^18^	126	- Confirmed the prognostic value of the WIfI classification in relation to risk of amputation and death in non-diabetic patients treated with endovascular techniques.
Ward et al.^19^	98	- Amputation rates increased as WIfI stage increased.
		- Revascularization significantly reduced the risk of amputation among patients at high risk of amputation at 1 year, classified by the WIfI.
Darling et al.^14^	992	- The study proposed two new scores to be used to complement or substitute the WIfI classification, the composite WIfI score and the mean WIfI score, because they were better predictors of the outcomes studied (mortality, risk of amputation, and RAS events), easier to administrate, and better for comparing results between patients.
		- The WIfI classification was a good predictor of major amputations and RAS events, but not of mortality. Only the new scores were consistent predictors of mortality in this study.
		- Patients revascularized for CLTI for the first time were at greater risk of a major amputation in the future, the higher their classification on any of the three scores used in the study.
Robinson et al.^20^	262	- Increases in WIfI stage were correlated with reductions in- AFS, but not with 1-year mortality.
Mathioudakis et al.^21^	279	-According to the study, “Among patients with DFU, the WIfI classification system correlated well with wound healing but was not associated with risk of major amputation at 1 year”.
		- Argues that a multidisciplinary approach to DFUs could help to reduction risk of amputation in patients at stage4.
Hicks et al.^22^		-The WIfI classification was capable of predicting healing of wounds at 1 year in patients with DFU. Mean healing time increased as WIfI stage increased.
Van Haelst et al.^23^	150	-In a subpopulation with CLTI, without revascularization options, the SVS WIfI classification correlated well with mortality, minor and major amputation, AFS, and wound healing.
		- The study suggested that stage 2 patients (WIfI 030) should be reallocated to stage 3 to better reflect the risk of amputation when revascularization is not an option.

SVS = Society for Vascular Surgery; WIfI = Wound, Ischemia, and foot Infection; AFS = amputation-free survival; CLI = critical limb ischemia; CLTI = chronic limb-threatening ischemia; RAS = reintervention, amputation, and stenosis; DFU = diabetic foot ulcers.

Limb salvage rate: amputation-free survival/risk of amputation at 1 year/major amputation

In 2014, Cull et al. 15 published a prospective study that analyzed 139 patients with CLI who underwent a lower limb revascularization procedure. The rates of % at stage 3; and 38% for limbs at stage 4. The authors of the study concluded that as the WIfI classification clinical stage increases, the risk of amputation at 1 year increases and AFS decreases. 15 In 2015, another group of researchers published a study in which they prospectively administered the SVS WIfI classification to 201 patients with CLI over a 2-year period. The AFS at 1 year rate was 100% for stages 1 and 2; 92% for stage 3; and 63% for stage 4. The limb salvage rate at each stage were 25%, 31%, 31%, and 13%, respectively. As in the earlier study, major amputation rates increased with each additional stage.^12^ Later studies also reported the WIfI classification’s capacity to predict risk of amputation at 1 year and to correlate well with AFS and, consequently, with limb salvage rates (Table 7).^16^^,^^18^^,^^19^ In general, the WIfI classification was correlated with risk of amputation at 1 year if predominantly administered to patients without diabetes who needed revascularization;^18^ to diabetic patients with wounds;^12^^,^^17^^,^^19^ only to patients treated with open or endovascular revascularization;^14^^,^^15^ or to patients treated with endovascular techniques.^16^

**Table 7:** Number of threatened limbs at each stage of the Wound, Ischemia, and foot Infection (WIfI) classification, with medians, weighted means, and 1-year amputation rates in parentheses.

**Study: nº of threatened limbs**	**Stage 1**	**Stage 2**	**Stage 3**	**Stage 4**
Cull et al.:^15^ 151	37 (3%)	63 (10%)	43 (23%)	8 (40%)
Zhan et al.:^12^ 201	39 (0%)	50 (0%)	53 (8%)	59 (64%)
Darling et al.:^16^ 551	5 (0%)	111 (10%)	222 (11%)	213 (24%)
Causey et al.:^17^ 160	21 (0%)	48 (8%)	42 (5%)	49 (20%)
Beropoulis et al.:^18^ 126	29 (0%)	42 (2%)	29 (3%)	26 (12%)
Ward et al.:^19^ 98	5 (0%)	21 (14%)	14 (21%)	58 (34%)
Darling et al.:^14^ 992	12 (0%)	293 (4%)	249 (4%)	438 (21%)
Robinson et al.:^20^ 262	48 (4%)	67 (16%)	64 (10%)	83 (22%)
Mathioudakis et al.:^21^ 279	95 (6.5%)	33 (6%)	87 (8%)	64(6%)
Van Haelst et al.:^23^ 150	15 (0%)	50 (16%)	32 (13%)	53 (42%)
N = 2,970 (weighted mean)	306 (3%)	778 (7.32%)	835 (8.61%)	1,051 (25%)
Median (1-year amputation rate, %)	0%	9%	9%	23%

However, some studies observed that major amputation rates at stage 2 were the same or even higher than rates at stage 3.^14^^,^^17^^,^^20^^,^^23^ Recently, a group of researchers from Johns Hopkins Hospital conducted a retrospective analysis of 217 patients with 279 limbs affected by DFU who were seen at their multidisciplinary clinic from 2012 to 2015 and found that the incidence of major amputation at 12 months was similar across WIfI classification stages. They concluded that this classification system was not predictive of risk of major amputation at 1 year for diabetic patients with DFU.^21^

### Multidisciplinary approaches

It has been suggested in the literature that care for the diabetic foot requires a multidisciplinary team, comprising, as a minimum, a vascular surgeon and a podiatrist (a medical professional who treats clinical and surgical conditions of the feet and lower limbs) specialized in the diabetic foot. This composition characterizes the “toe and flow” model and more specialists should be added to the team as needed.^24^ Studies of the WIfI classification have suggested that a multidisciplinary approach could be beneficial not just for the diabetic foot, but also for preservation of the threatened limb in general, including for those classified at the higher stages of the SVS WIfI classification.^20^ One of the outcomes observed is a reduction in the risk of amputation.^21^ However, while limb salvage outcomes are excellent, the total cost of multidisciplinary treatment appears to be very high, especially for patients with advance disease (WIfI stages 3 and 4).^25^

### Revascularization

One of the primary outcomes expected from creation of the WIfI classification was the capacity to predict the need for revascularization of the threatened limb. In the majority of studies, revascularizations, whether open or by endovascular procedures, were more common as the WIfI stage increased.^17^^,^^20^^,^^21^ At higher classification stages, revascularization proved beneficial for reducing the risk of amputation by 25%.^17^ In a study by Robinson et al.,^20^ it was observed that the primary reason for use of revascularization was the degree of ischemia and that there was a statistically significant revascularization benefit for patients with grade 3 ischemia, compared with a strong trend to benefit in patients at grades 1 and 2. These results confirm the recommendations of the SVS WIfI classification expert panel, who proposed that revascularization would be highly beneficial/necessary for almost all patients with grade 3 ischemia and selected patients with grade 2.^20^

In a 2015 study, revascularization resulted in a reduction in the time taken for wounds to heal among patients at stage 3, primarily due to the characteristics of the wound and the degree of ischemia.^12^ However, in the same study, just 2 of the 89 patients (2%) at stages 1 and 2 underwent revascularization, none of the patients at stages 1 and 2 needed amputation within 1 year, and AFS was 100% for both groups.^12^ This encouraging rate of AFS at the less severe stages of CLI, even without revascularization, raises questions about the benefits of the procedure.^18^ Furthermore, there was no significant difference in amputation rates between limbs classified as high risk of amputation, irrespective of whether or not they were revascularized. This suggests that there may be factors associated with amputation that are not included in the WIfI classification and that some patients classified as at high risk of amputation may not benefit from revascularization.^19^

In 2018, a study analyzed the relationship between WIfI classification stage and clinical outcomes in a subpopulation with chronic limb-threatening ischemia (CLTI) with no options for revascularization (i.e., with distal lesions unsuitable for revascularization, with no available autologous venous material, and with no endovascular options). The SVS WifI classification was correlated with short and medium-term clinical outcomes, including mortality, minor and major amputations, AFS, and wound healing. The data suggest that prognosis is poor for patients with classical ischemic pain, without wounds or infection (W0-I3-fI0), and no options for revascularization. In view of this, the study proposed reallocating this subpopulation of patients with ischemic pain at rest from stage 2 to stage 3 of the WIfI classification to better reflect the risk of amputation in the absence of successful revascularization.^23^

### Wound healing

A prospective study for early validation of the WIfI classification discovered that the classification’s clinical stages were also well correlated with rates of wound healing.^15^ Since then, several research teams have found that the higher the WIfI classification clinical stage, the worse the wound healing^15^^,^^16^^,^^20^^,^^23^ (for example: incomplete healing) or the longer the time needed for healing.^12^^,^^21^^,^^22^ Higher wound grades and presence of infection were individually associated with incomplete healing.^16^ In a study by Van Haelst et al.,^23^ just 19% of wounds healed in patients at stage 4 of the SVS WIfI classification, whereas all wounds healed in patients at stage 1.

### Global survival/mortality

In the majority of studies about the WIfI classification, it did not prove to be a good predictor of mortality or did not have a good correlation with 1-year survival/mortality.^14^^,^^16^^,^^17^^,^^20^

Darling et al.^14^ proposed two new scores: a mean WIfI score, which stratifies TLL by grades 0-3 and enables inclusion of limbs that do not have one of the parameters of the SVS WifI classification. (wound, ischemia, or foot infection); and a composite WIfI score, which ranges from 0 to 9 and weights all WIfI variables equally. In that study, 903 patients, with 992 limbs treated with revascularization for the first time, were divided into three cohorts: (1) all patients; (2) patients who only underwent conventional surgery; and (3) patients who only underwent an endovascular procedure. The new scores were the only consistent predictors of mortality in the three cohorts, while the SVS WifI classification was not associated with mortality in any of them.^14^^,^^26^

However, Beropoulis et al.^18^ validated the prognostic capacity of WIfI classification clinical stages from 1 to 4 with relation to amputation and mortality in a highly specific subset: non-diabetic patients treated with endovascular techniques. Additionally, Novak et al.^27^ confirmed that wound grade was the WIfI classification factor most predictive of patient survival.

### Re-staging after intervention

An early validation of the WIfI classification had already suggested its capacity to predict clinical outcomes after revascularization.^15^ Leithead et al.^28^ found that in addition to simply stratifying before the intervention, re-staging with the SVS WIfI classification is an important tool for preventing limb loss and assessing the efficacy of the intervention.

### Reintervention, amputation, and stenosis events

Darling et al.^14^^,^^16^ conducted two studies of the WIfI classification, suggesting that among patients who undergo lower limb revascularization, whether open or endovascular, an increase of one grade in SVS WIfI classification clinical stage was associated with increased risk of reintervention, amputation, and stenosis (RAS) events. The studies also supported the capacity of the SVS WIfI classification system to predict the risk of amputation at 1 year and the rates of wound healing in patients with CLI who underwent below-the-knee endovascular revascularization procedures. Another retrospective analysis of 302 non-diabetic patients who underwent vascular interventions from 2013 to 2014 also found that, in this subpopulation, reintervention occurs more frequently in the subset classified at clinical stage 3.^18^

It is important to point out that the SVS WIfI classification is just one of three components necessary to create a TLL treatment sequence. A stratification that covers patient comorbidities and another that assesses the anatomic pattern of the disease and its severity are also necessary.^1^^,^^12^ The SVS, the European Society for Vascular Surgery, and the World Federation of Vascular Societies jointly created the Global Vascular Guidelines: new guidelines for management of CLTI, that were finalized and published in 2019.^29^ In addition to being endorsed by these guidelines, the WIfI classification will also be validated in large studies such as Best Endovascular vs. Best Surgical Therapy in Patients with Critical Limb Ischemia (BEST- CLI), which is already in the final stages of preparation.^30^

## CONCLUSIONS

The SVS WIfI classification system is the topic of ongoing debate when the subject is improving treatment for patients with TLL. The majority of validation studies of this classification demonstrate that it is associated with factors linked to limb salvage, such as rates of minor and major amputations, AFS, prediction of RAS events, and wound healing. Concerted efforts are still needed to optimize treatment for patients with TLL, improving the ways that they are analyzed and stratified and, as a consequence, managed clinically.
