# Supplementary material for: WIfI classification: the Society for Vascular Surgery lower extremity threatened limb classification system, a literature review
Source: J Vasc Bras. 2020 May 8;19:e20190070. doi: 10.1590/1677-5449.190070 (PMC8202158; doi:10.1590/1677-5449.190070)
Supplement: Translation

# Translation: Classificação WifI: o novo sistema de classificação da *Society for Vascular Surgery* para membros inferiores ameaçados, uma revisão de literatura

**Authors:** Lorena de Oliveira Cerqueira, Eliud Garcia Duarte, André Luis de Souza Barros, José Roberto Cerqueira, Walter Júnior Boim de Araújo

**Affiliations:** 1 Universidade Vila Velha – UVV, Cirurgia Vascular, Vila Velha, ES, Brasil.; 2 Centro de Atuação Precoce em Úlceras Vasculares e Complicações do Pé Diabético – PROPÉ, Departamento de Cirurgia Vascular, Vila Velha, ES, Brasil.; 3 Hospital Estadual de Urgência e Emergência do Espírito Santo – HEUE, Departamento de Cirurgia Vascular, Vila Velha, ES, Brasil.; 4 Hospital Santa Mônica de Vila Velha, Serviço de Cirurgia Vascular e Endovascular, Vila Velha, ES, Brasil.; 5 Universidade Federal do Paraná – UFPR, Hospital de Clínicas, Serviço de Angiorradiologia e Cirurgia Endovascular, Curitiba, PR, Brasil.; 6 Hospital Angelina Caron, Departamento de Residência Médica em Cirurgia Vascular e Endovascular, Curitiba, PR, Brasil.

**Keywords:** pé diabético, úlcera do pé, isquemia, infecção, amputação, mortalidade

## Abstract

A *Society for Vascular Surgery* propôs nova classificação para o membro inferior ameaçado, baseada nos três principais fatores influenciadores do risco de amputação do membro: ferida (*Wound*, W), isquemia (*Ischemia*, I) e infecção do pé (*foot Infection*, fI): a classificação WIfI. Esta abrange também os diabéticos, anteriormente excluídos do conceito de isquemia crítica do membro devido a seu quadro clínico complexo. O objetivo da classificação era fornecer estratificação de risco precisa e precoce ao paciente com membro inferior ameaçado; auxiliar no manejo clínico, permitindo comparar terapias alternativas; e predizer o risco de amputação em 1 ano e a necessidade de revascularização. O objetivo deste estudo é reunir os principais pontos abordados sobre a classificação WIfI no meio científico. A maior parte dos estudos de validação da classificação demonstram sua associação à predição de salvamento do membro, eventos de reintervenção, amputação e estenose, taxas de amputação maior e menor, sobrevida livre de amputação, e cicatrização de feridas.

## INTRODUÇÃO

Desde 1982, quando o primeiro conceito de isquemia crítica do membro (ICM) surgiu, o mundo passou por inúmeras mudanças, incluindo avanços na medicina e alterações no perfil dos pacientes. Originalmente, a ICM foi definida como pressão arterial sistólica do tornozelo (*ankle pressure*, AP) < 40 mmHg na presença de dor em repouso e < 60 mmHg em vigência de necrose tecidual^1^^,^^2^. Os pacientes diabéticos foram excluídos desse conceito, como enfatizado no final do documento publicado em 1982:

Foi geralmente aceito que pacientes diabéticos que têm um quadro clínico variado de neuropatia, isquemia e sepse tornam a definição ainda mais difícil e é desejável que esses pacientes sejam excluídos... Ou devam ser claramente definidos como uma categoria separada para permitir a análise dos resultados nos pacientes não diabéticos^3^ (p. S2-3).

O objetivo do conceito inicial era, portanto, delimitar um grupo de pacientes sem diabetes e com um membro inferior ameaçado (MIA) por isquemia crônica que, sem revascularização, seria inevitavelmente perdido^3^.

Em 2016, em relatório publicado em razão do Dia Mundial da Saúde, a Organização Mundial da Saúde (OMS) definiu o diabetes como sendo uma epidemia global. Segundo o documento, desde 1980 o número de adultos vivendo com o diabetes teria quadruplicado e alcançado os 422 milhões em 2014, refletindo um aumento nos fatores de risco associados, como sobrepeso e obesidade^4^. Com o maior número de diabéticos, veio também o aumento da incidência de úlceras do pé diabético (UPD) e da doença arterial periférica nesses pacientes^5^. Ou seja, o perfil do paciente mais comum que, há 40 anos, era um fumante, não diabético, do sexo masculino e com aterosclerose, mudou. Além disso, vivemos uma crescente modernização das técnicas de revascularização e de outras abordagens de tratamento, que são apresentadas ao cirurgião vascular como terapias alternativas e que precisam ser comparadas e escolhidas individualmente^2^.

## OS SISTEMAS ANTERIORES E A NECESSIDADE DE UMA NOVA CLASSIFICAÇÃO

Já é validado que, na complexidade de um MIA, seja ele de um paciente com diabetes ou não, a perfusão é apenas um determinante do resultado, sendo que as características da ferida, além da presença e da gravidade da infecção, são também fatores de grande impacto no risco de amputação do membro^6^^,^^7^.

Diante disso, são encontrados alguns problemas no conceito de ICM e nas classificações atuais para MIAs. Em primeiro lugar, a fundamentação e a história natural do conceito de ICM determinavam que existiria um valor crítico abaixo do qual a perfusão do membro seria inadequada e que, sem a revascularização, o membro seria fatalmente amputado. No entanto, estudos já demonstraram que pacientes com ICM podem manter seus membros, mesmo sem serem submetidos a revascularização^1^^,^^2^. Um exemplo é o estudo de Elgzyri et al.^8^, na Suécia, que avaliou 602 pacientes com UPD, pressão arterial sistólica do dedo do pé (*toe pressure*, TP) < 45 mmHg ou AP < 80 mmHg, e que não foram revascularizados. Esse estudo relatou que 50% obtiveram uma evolução favorável apenas com tratamento de feridas ou amputação menor; 17% melhoraram após amputação maior; e 33% morreram com membros intactos, mas com feridas não cicatrizadas.

Em segundo lugar, o conceito original de ICM não inclui os diabéticos.

Em terceiro lugar, os sistemas de classificação preexistentes para membros ameaçados são limitados, pois geralmente não abordam todos os três pilares (ferida, isquemia e infecção) da extremidade em risco de amputação ou não diferenciam úlcera de gangrena, não abrangendo o MIA em toda a sua heterogeneidade de causas e apresentações clínicas. Por exemplo, as classificações de Rutherford et al.^9^ e Fontaine et al.^10^ (Tabela 1) baseiam-se principalmente no grau de isquemia, e a classificação de Wagner^11^ (Tabela 2), ainda amplamente utilizada para as feridas do pé diabético, não auxilia muito na diferenciação entre causas de gangrena isquêmicas e infecciosas^12^. Enquanto isso, o *Inter-Society Consensus for the Management of Peripheral Arterial Disease* (TASC II), publicado em 2007, dizia que 20% dos pacientes com ICM crônica morriam no primeiro ano de apresentação clínica da doença; 10% dos pacientes amputados em níveis abaixo do joelho morriam no período perioperatório; e, após 2 anos da cirurgia, 30% estavam mortos^13^. Ou seja, havia a necessidade urgente de se criar um sistema de estadiamento que fornecesse estratificação de risco precisa e precoce dos pacientes com relação à história natural da doença, abrangendo os três principais fatores que influenciam o risco de amputação do membro e o manejo clínico. Além disso, havia carência de uma ferramenta que permitisse ao profissional uma comparação significativa entre as diferentes terapias e que o ajudasse na tomada de decisão clínica^1^^,^^14^.

**Tabela 1:** Classificações de Fontaine et al.^10^ e Rutherford et al.^9^ para doença arterial periférica.

**Fontaine et al.** **^10^**		**Rutherford et al.** **^9^**		
**Estágio**	**Clínica**	**Grau**	**Categoria**	**Clínica**
I	Assintomático	0	0	Assintomático
IIa	Claudicação leve	I	1	Claudicação leve
IIb	Cladicação moderada a severa	I	2	Claudicação moderada
III	Dor isquêmica em repouso	I	3	Claudicação grave
IV	Gangrena ou ulceração	II	4	Dor isquêmica em repouso
		III	5	Lesão tecidual menor
		III	6	Lesão tecidual maior

**Tabela 2:** Classificação de Wagner^11^ para as lesões do pé diabético.

**Grau**	**Características**
0	Pé de risco, sem úlcera evidente, com calosidades grossas e cabeças metatársicas proeminentes, dedos em garra ou outras anormalidades ósseas
1	Úlcera superficial não infectada
2	Úlcera profunda sem envolvimento ósseo
3	Úlcera profunda com formação de abscesso ou envolvimento ósseo
4	Gangrena localizada em parte do pé
5	Gangrena extensa de todo o pé

Pensando nisso, a *Society for Vascular Surgery* (SVS) desenvolveu um novo sistema de classificação que dispensa o termo ICM, baseando-se nas características da ferida (*wound*, W), no grau de isquemia (*ischemia*, I), e na presença e gravidade da infecção (*foot infection*, fI): a classificação *Wound, Ischemia and foot Infection* da SVS, ou classificação WIfI^1^^,^^2^.

O objetivo deste estudo é reunir os principais pontos abordados sobre a classificação WIfI em meio à comunidade científica, desde o artigo da SVS, publicado em 2014^1^, até a presente data.

## A CLASSIFICAÇÃO WIFI DA SVS

O sistema de classificação da SVS, desenvolvido em 2013 e proposto em publicação pela referida sociedade em 2014, aborda os três principais parâmetros que colocam um membro em risco de amputação: ferida, isquemia e infecção do pé^1^. A classificação WIfI da SVS atribui a cada letra ou parâmetro da sigla uma escala de 4 graus, que vai de 0 a 3, onde 0 representa ausente, 1 leve, 2 moderado, e 3 grave (Tabela 3). Após esse escalonamento, a pontuação atribuída a cada letra é combinada e analisada em duas tabelas: uma de estimativa do risco de amputação em 1 ano e outra de estimativa de necessidade/benefício com a revascularização (Tabelas 4 e 5). Com base nos resultados encontrados, o membro é classificado para risco de amputação e necessidade de revascularização nos estágios clínicos 1, 2, 3 ou 4 da classificação: muito baixo, baixo, moderado e alto risco de amputação ou benefício com a revascularização, respectivamente (Tabela 4 e 5). O estágio 5 representa um membro irrecuperável, mesmo com revascularização. Os estágios foram elaborados por um rol de especialistas que, para chegar a um consenso, categorizou cada uma das 64 combinações possíveis da tabela seguindo o método Delphi^1^.

**Tabela 3:** A classificação WIfI para membros inferiores ameaçados: avaliação do risco de amputação^1^.

**Componente**	**Graus**	**Descrição**		
Ferida (**W)**	**0**	Sem úlcera ou gangrena (dor isquêmica em repouso)		
	**1**	Úlcera pequena ou superficial em perna ou pé, sem gangrena (ADS ou CP)		
	**2**	Úlcera profunda com exposição de osso, articulação ou tendão ± gangrena limitada a pododáctilos (MAD ou TMA padrão ± CP)		
	**3**	Úlcera profunda e extensa envolvendo antepé e/ou mediopé ± envolvimento do calcâneo ± gangrena extensa (RC do pé ou TMA não tradicional)		
Isquemia (**I)**	**0**	**ITB**	**PAS do tornozelo**	**TP, TcPO_2_**
	**1**	≥ 0,80	> 100 mmHg	≥ 60 mmHg
	**2**	0,6-0,79	70-100 mmHg	40-59 mmHg
	**3**	0,4-0,59 ≤ 0.39	50-70 mmHg < 50 mmHg	30-39 mmHg < 30 mmHg
Infecção do pé (**fI**)	**0**	Não infectado		
	**1**	Infecção local leve, envolvendo apenas pele e subcutâneo, eritema > 0,5 e ≤ 2 cm		
	**2**	Infecção local moderada, com eritema > 2 cm ou envolvendo estruturas mais profundas		
	**3**	Infecção local grave com os sinais de SIRS		

WIfI = *Wound, Isquemia and Foot infection*; ADS = amputação digital simples; CP = cobertura da pele; MAD = múltiplas amputações digitais; TMA = amputação transmetatársica; RC = reconstrução complexa; ITB = índice tornozelo-braquial; PAS = pressão arterial sistólica; TP = *toe pressure* (PAS do dedo do pé); TCPO_2_ = *transcutaneous oxygen pressure* (pressão transcutânea de oxigênio); SIRS = *systemic inflammatory response syndrome* (síndrome da resposta inflamatória sistêmica).

**Tabela 4:** Estimativa do risco de amputação em 1 ano, de acordo com os estágios clínicos da classificação WIfI, propostos pelo rol de especialistas^1^.

	**Isquemia 0**				**Isquemia 1**				**Isquemia 2**				**Isquemia 3**			
Ferida 0	VL	VL	L	M	VL	L	M	H	L	L	M	H	L	M	M	H
Ferida 1	VL	VL	L	M	VL	L	M	H	L	M	H	H	M	M	H	H
Ferida 2	L	L	M	H	M	M	H	H	M	H	H	H	H	H	H	H
Ferida 3	M	M	H	H	H	H	H	H	H	H	H	H	H	H	H	H
	fI 0	fI 1	fI 2	fI 3	fI 0	fI 1	fI 2	fI 3	fI 0	fI 1	fI 2	fI 3	fI 0	fI 1	fI 2	fI 3

VL = *very low* (muito baixo); L = *low* (baixo); M = *moderate* (moderado); H = *high* (alto); fI = *foot infection* (infecção do pé); WIfI = *Wound, Isquemia and Foot infection*.

**Tabela 5:** Estimativa da necessidade/benefício da revascularização, de acordo com os estágios clínicos da classificação WIfI, propostos pelo rol de especialistas. A infecção precisa estar controlada^1^.

	**Isquemia 0**				**Isquemia 1**				**Isquemia 2**				**Isquemia 3**			
Ferida 0	VL	VL	VL	VL	VL	L	L	M	L	L	M	M	M	H	H	H
Ferida 1	VL	VL	VL	VL	L	M	M	M	M	H	H	H	H	H	H	H
Ferida 2	VL	VL	VL	VL	M	M	H	H	H	H	H	H	H	H	H	H
Ferida 3	VL	VL	VL	VL	M	M	M	H	H	H	H	H	H	H	H	H
	fI 0	fI 1	fI 2	fI 3	fI 0	fI 1	fI 2	fI 3	fI 0	fI 1	fI 2	fI 3	fI 0	fI 1	fI 2	fI 3

VL = *very low* (muito baixo); L = *low* (baixo); M = *moderate* (moderado); H = *high* (alto); fI = *foot infection* (infecção do pé); WIfI = *Wound, Isquemia and Foot infection*.

Esse sistema foi criado para definir precisamente o ônus da doença, e não com o intuito de ditar o método de tratamento, uma vez que as modalidades terapêuticas continuam a evoluir. Assim, destina-se a ser para o MIA um análogo do que a classificação Tumor, Nódulo, Metástase (TNM) é para o câncer, auxiliando no manejo clínico e permitindo comparações entre grupos semelhantes de pacientes e entre terapias alternativas^1^^,^^2^^,^^15^.

A população-alvo para esse sistema inclui qualquer paciente com: dor isquêmica em repouso, tipicamente em antepé; estudos hemodinâmicos objetivos confirmatórios (índice tornozelo-braquial [ITB] < 0,40, AP < 50 mmHg, TP < 30 mmHg e/ou pressão transcutânea de O_2_ [*transcutaneous oxygen pressure*, TcPO_2_] < 20 mmHg); UPD; ulceração do pé ou ferida não cicatrizada em membro inferior (MI) com duração ≥ 2 semanas e/ou gangrena em MI ou pé. As seguintes condições são excluídas: pacientes com úlceras venosas puras; feridas relacionadas a condições não ateroscleróticas (por exemplo: vasculite, doença vascular do colágeno, doença de Buerger, radiação); isquemia aguda do membro; *trash foot* agudo ou isquemia por êmbolo; trauma agudo/mutilação da extremidade^1^.

### W: ferida/categoria clínica

Na classificação WIfI da SVS, a ferida é classificada com base no tamanho, na profundidade, na gravidade e, diferentemente das classificações anteriores, na complexidade do procedimento mais provavelmente necessário à sua cicatrização. Além disso, a gangrena é incluída e estratificada por extensão (Tabela 3)^1^^,^^2^.

### I: isquemia

O grau de isquemia pode ser medido pelo ITB, que, se for ≥ 0,80, é classificado como grau 0. Se o ITB for incompressível (> 1,3), a TP ou a TcPO_2_ devem ser medidas. Medir a TP é obrigatório em todos os pacientes portadores de diabetes, pois as medidas do ITB podem estar falsamente elevadas devido a calcificações. Se o ITB e a TP resultarem em valores diferentes, a TP será o principal determinante do grau de isquemia (Tabela 3)^1^^,^^2^.

### fI: infecção do pé

A WIfI classifica a presença e a gravidade da infecção, levando em consideração os sistemas de classificação anteriores para pé diabético *Perfusion, Extent, Depth, Infection and Sensation* (PEDIS) e *Infectious Diseases Society of America* (IDSA). Se o fI for considerado grau 3 ou grave, o paciente já apresenta sinais sistêmicos da infecção (Tabela 3)^1^^,^^2^.

Exemplificando: um homem de 56 anos, não portador de diabetes, apresenta dor isquêmica em repouso, mas sem ferida. O ITB é de 0,36 e não há sinais de infecção local ou sistêmica. Ele poderia ser classificado como ferida 0, isquemia 3 e infecção do pé 0, ou WIfI 030. Seu estágio clínico seria 2 (baixo risco de amputação em 1 ano), e o benefício com a revascularização seria moderado (Tabelas 4 e 5).

## VALIDAÇÃO DO SISTEMA: CORRELAÇÕES COM DESFECHOS ESPERADOS E NÃO ESPERADOS

Desde a publicação inicial de Mills et al.^1^, vários estudos importantes foram publicados para testar e validar o sistema de classificação WIfI da SVS (Tabela 6). Abaixo apresentaremos os principais estudos já publicados até hoje que discutem a validação do sistema e apontam suas vantagens, limitações e desafios para o futuro. Dividimos os desfechos estudados de maneira didática. Porém, na maioria dos trabalhos, alguns desfechos estão fortemente correlacionados.

**Tabela 6:** Principais estudos em que se baseou este trabalho, publicados para testar e validar a classificação WIfI da SVS, e suas conclusões.

**Estudo**	**Nº de membros ameaçados**	**Conclusões e tópicos importantes**
Cull et al.^15^	151	- Quanto maior é o estágio clínico WIfI, maior é o risco de amputação em 1 ano e pior é a cicatrização das feridas. - O diabetes foi independentemente associado a um maior risco de amputação em 1 ano.
Zhan et al.^12^	201	- À medida que o estágio clínico WIfI progride, o risco de amputação maior aumenta, a SLA em 1 ano diminui, e o tempo de cicatrização se prolonga. - A revascularização acelerou o tempo de cicatrização de feridas em pacientes estágio 3.
Darling et al.^16^	551	- A classificação WIfI foi capaz de prever risco de amputação em 1 ano e taxa de cicatrização de feridas em pacientes com ICM já submetidos a tratamento infrapoplíteo, por via endovascular.
Causey et al.^17^	160	- O estudo defende o uso da classificação WIfI e do*Project or Ex-Vivo vein graft Engineering via Transfection III* (PIII) como ferramentas complementares no manejo da CLTI. - A classificação WIfI correlacionou-se bem com o tempo de internação e com eventos adversos graves dos membros, como amputação, a médio prazo. - As taxas de revascularização aumentaram com o estágio WIfI. Sugere-se que a classificação é capaz de prever o benefício do paciente com a revascularização.
Beropoulis et al.^18^	126	- Confirmou-se o valor prognóstico da classificação WIfI em relação ao risco de amputação e à morte em pacientes não diabéticos tratados por via endovascular.
Ward et al.^19^	98	- As taxas de amputação aumentaram com a elevação do estágio WIfI. - A revascularização reduziu significativamente o risco de amputação entre pacientes com alto risco de amputação em 1 ano, classificados pela WIfI.
Darling et al.^14^	992	- O estudo propõe dois novos escores para serem usados complementarmente ou em substituição à classificação WIfI: o escore WIfI composto e o escore WIfI médio, pois eles seriam melhores preditores dos desfechos estudados (mortalidade, risco de amputação e de eventos RAS), mais fáceis de aplicar e melhores para comparar resultados entre pacientes. - A classificação WIfI foi boa preditora de grandes amputações e de eventos RAS, mas não de mortalidade. Apenas os novos escores foram preditores consistentes de mortalidade, nesse estudo. - Pacientes revascularizados pela primeira vez para CLTI tiveram maior risco de sofrer amputação maior no futuro quanto maior fosse sua classificação em qualquer um dos três escores utilizados no estudo.
Robinson et al.^20^	262	- O aumento do estágio WIfI correlacionou-se com diminuição da SLA, mas não com a mortalidade em 1 ano.
Mathioudakis et al.^21^	279	- Segundo o estudo, “entre os pacientes com UPD, o sistema de classificação WIfI correlacionou-se bem com a cicatrização de feridas, mas não foi associado ao risco de amputação maior em 1 ano”. - Defende-se que a abordagem multidisciplinar às UPDs pode auxiliar na redução do risco de amputação em pacientes estágio 4.
Hicks et al.^22^		-A classificação WIfI foi capaz de prever a cicatrização de feridas em 1 ano entre pacientes com UPD. O tempo médio de cicatrização aumentou com o aumento do estágio WIfI.
Van Haelst et al.^23^	150	- Em uma subpopulação com CLTI, sem opção de revascularização, a classificação WIfI da SVS correlacionou-se bem com mortalidade, amputação maior e menor, SLA e cicatrização de feridas. - O estudo sugere que os pacientes estágio 2 (WIfI 030) sejam realocados para o estágio 3, para melhor refletir o risco de amputação na ausência de revascularização.

SVS = *Society for Vascular Surgery*; WIfI = *Wound, Isquemia and foot Infection*; SLA = sobrevida livre de amputação; ICM = isquemia crítica do membro; CLTI = *chronic limb-threatening ischemia* (isquemia crônica ameaçadora dos membros); RAS = reintervenção, amputação e estenose; UPD = úlceras do pé diabético.

Taxa de salvamento do membro: sobrevida livre de amputação/ risco de amputação em 1 ano/ amputação maior

Em 2014, Cull et al.^15^ publicaram um estudo prospectivo que analisou 139 pacientes com ICM submetidos a algum procedimento de revascularização do MI. As taxas de amputação maior relatadas aumentaram a cada estágio da classificação WifI da SVS. A sobrevida livre de amputação (SLA) em 1 ano foram de 86% nos membros do estágio 1; 83% naqueles do estágio 2; 70% nos do estágio 3; e 38% nos membros do estágio 4. Os autores do estudo concluíram que, com o avanço do estágio clínico na classificação WIfI, maior é o risco de amputação em 1 ano e menor é a SLA^15^. Em 2015, outro grupo de pesquisadores publicou um estudo no qual aplicou prospectivamente a classificação WIfI da SVS a 201 pacientes com ICM por um período de 2 anos. A SLA em 1 ano foi de 100% para os estágios 1 e 2; 92% para o estágio 3; e 63% para o estágio 4. As taxas de salvamento do membro em cada estágio foram de 25%, 31%, 31% e 13%, respectivamente. As taxas de amputação maior, assim como no estudo anterior, cresceram a cada estágio^12^. Estudos subsequentes também reportaram a capacidade da classificação WIfI de prever o risco de amputação em 1 ano e de se correlacionar bem com a SLA e, consequentemente, com as taxas de salvamento do membro (Tabela 7)^16^^,^^18^^,^^19^. Num panorama geral, a classificação WIfI se correlacionou com o risco de amputação em 1 ano se aplicada predominantemente a pacientes não diabéticos que necessitam de revascularização^18^; a pacientes diabéticos com feridas^12^^,^^17^^,^^19^; apenas a pacientes submetidos a revascularização aberta ou endovascular^14^^,^^15^; ou a pacientes tratados por terapia endovascular^16^.

**Tabela 7:** Número de membros ameaçados em cada estágio da classificação *Wound, Ischemia and foot Infection* (WIfI), com medianas, médias ponderadas e taxas de amputação em 1 ano entre parênteses.

**Estudo: nº de membros ameaçados**	**Estágio 1**	**Estágio 2**	**Estágio 3**	**Estágio 4**
Cull et al.^15^: 151	37 (3%)	63 (10%)	43 (23%)	8 (40%)
Zhan et al.^12^: 201	39 (0%)	50 (0%)	53 (8%)	59 (64%)
Darling et al.^16^: 551	5 (0%)	111 (10%)	222 (11%)	213 (24%)
Causey et al.^17^: 160	21 (0%)	48 (8%)	42 (5%)	49 (20%)
Beropoulis et al.^18^: 126	29 (0%)	42 (2%)	29 (3%)	26 (12%)
Ward et al.^19^: 98	5 (0%)	21 (14%)	14 (21%)	58 (34%)
Darling et al.^14^: 992	12 (0%)	293 (4%)	249 (4%)	438 (21%)
Robinson et al.^20^: 262	48 (4%)	67 (16%)	64 (10%)	83 (22%)
Mathioudakis et al.^21^: 279	95 (6,5%)	33 (6%)	87 (8%)	64(6%)
Van Haelst et al.^23^: 150	15 (0%)	50 (16%)	32 (13%)	53 (42%)
N = 2.970 (média ponderada)	306 (3%)	778 (7,32%)	835 (8,61%)	1.051 (25%)
Mediana (taxa de amputação em 1 ano, %)	0%	9%	9%	23%

No entanto, alguns trabalhos observaram que as taxas de amputação maior no estágio 2 foram as mesmas ou até mais altas que no estágio 3^14^^,^^17^^,^^20^^,^^23^. Recentemente, um grupo de pesquisadores do Johns Hopkins Hospital fez uma análise retrospectiva de 217 pacientes com 279 membros afetados por UPD que compareceram em sua clínica multidisciplinar entre 2012 e 2015 e percebeu que a incidência de amputação maior em 12 meses foi semelhante entre os estágios da classificação WIfI. Concluíram que esse sistema de classificação não foi preditivo para o risco de amputação maior em 1 ano entre os pacientes diabéticos com UPD^21^.

### Abordagem multidisciplinar

Já foi sugerido na literatura que o cuidado ao pé diabético requer atendimento por uma equipe multidisciplinar, composta minimamente por um cirurgião vascular e um *podiatrist* (profissional médico que trata de condições clínicas e cirúrgicas dos pés e membros inferiores) especializado em pé diabético. Essa composição caracteriza o modelo *toe and flow* (*toe =* pé, *podiatrist* e *flow =* fluxo, cirurgião vascular), e mais especialistas devem ser incluídos à equipe, à medida que se fizerem necessários^24^. Diante disso, os estudos sobre a classificação WIfI sugeriram que a abordagem multidisciplinar pode ser benéfica não só para o pé diabético, mas também para a preservação do membro ameaçado no geral, inclusive para aqueles classificados nos estágios mais avançados da classificação WIfI da SVS^20^. Um dos desfechos observados é a redução do risco de amputação^21^. No entanto, embora os desfechos para o salvamento de membros sejam excelentes, o custo total do tratamento multidisciplinar, especialmente para pacientes com doença avançada (estágios WIfI 3 e 4) parece ser consideravelmente alto^25^.

### Revascularização

Entre os desfechos primários esperados na criação da classificação WIfI estava a capacidade de predizer a necessidade de revascularização para o membro ameaçado. Na maioria dos estudos, as revascularizações, abertas ou por procedimento endovascular, foram mais comuns com o aumento do estágio WIfI^17^^,^^20^^,^^21^. Nos estágios mais altos da classificação, a revascularização mostrou-se benéfica ao reduzir o risco de amputação em 25%^17^. No estudo de Robinson et al.^20^, foi observado que o uso da revascularização teve como motivo principal o grau de isquemia e que houve um benefício estatisticamente significativo para a revascularização em pacientes com isquemia grau 3, comparada à forte tendência de benefício em pacientes dos graus 1 e 2. Esses resultados confirmam as recomendações do rol de especialistas da classificação WIfI da SVS, que propuseram que a revascularização seria altamente benéfica/necessária a quase todos os pacientes com isquemia grau 3 e a pacientes selecionados de grau 2^20^.

Em um estudo de 2015, a revascularização resultou em aceleração do tempo de cicatrização das feridas em pacientes no estágio 3, principalmente devido às características da ferida e ao grau de isquemia^12^. No entanto, nesse mesmo estudo, apenas 2 dos 89 pacientes (2%) nos estágios 1 e 2 foram submetidos a revascularização, nenhum paciente dos estágios 1 e 2 necessitou de amputação em 1 ano, e a SLA foi de 100% para ambos^12^. Essa taxa de SLA encorajadora nos estágios menos graves de ICM, mesmo sem revascularização, questiona o benefício desse procedimento^18^. Além disso, não houve diferença significativa nas taxas de amputação entre os membros classificados como de alto risco para amputação, independentemente de terem sido revascularizados ou não. Isso sugere que possam existir fatores associados à amputação que não são abrangidos pela classificação WIfI e que alguns pacientes classificados como de alto risco para amputação podem não se beneficiar da revascularização^19^.

Em 2018, um trabalho avaliou a relação entre o estágio na classificação WIfI e os desfechos clínicos em uma subpopulação com isquemia crônica ameaçadora dos membros (*chronic limb-threatening ischemia*, CLTI) sem opção de revascularização (isto é, com lesões distais não passíveis de revascularização, nenhum material venoso autólogo disponível e ausência de opções endovasculares). A classificação WifI da SVS correlacionou-se com desfechos clínicos de curto e médio prazo, incluindo mortalidade, amputação maior e menor, SLA e cicatrização de feridas. Os dados sugerem que o prognóstico é ruim para pacientes com dor isquêmica clássica, sem feridas ou infecção (W0-I3-fI0) e que não possuem opções de revascularização. Devido a isso, o estudo propõe realocar essa subpopulação de dor isquêmica em repouso do estágio 2 para o estágio 3 da classificação WIfI, para melhor refletir o risco de amputação na ausência de revascularização bem-sucedida^23^.

### Cicatrização de feridas

Um estudo prospectivo para validação precoce da classificação WIfI descobriu que os estágios clínicos da classificação também se correlacionavam muito bem com as taxas de cicatrização de feridas^15^. A partir de então, diversos grupos de pesquisadores constataram que, quanto maior é o estágio clínico na classificação WIfI, pior é a cicatrização das feridas^15^^,^^16^^,^^20^^,^^23^ (por exemplo: cicatrização incompleta) ou mais tempo elas demoram para cicatrizar^12^^,^^21^^,^^22^. O aumento do grau de ferida e a presença de infecção foram individualmente associados à cicatrização incompleta^16^. No estudo de Van Haelst et al.^23^, apenas 19% das feridas cicatrizaram em pacientes no estágio 4 da classificação WIfI da SVS, enquanto todas as feridas em estágio 1 cicatrizaram.

### Sobrevida global/mortalidade

Na maioria dos estudos sobre a classificação WIfI, esta não se mostrou boa preditora de mortalidade ou não obteve boa correlação com sobrevida/mortalidade em 1 ano^14^^,^^16^^,^^17^^,^^20^.

Darling et al.^14^ propuseram dois novos escores: o escore médio WIfI, que estratifica o MIA em graus 0-3 e permite incorporar membros que não possuem algum dos parâmetros da classificação WifI da SVS (ferida, isquemia ou infecção do pé); e o escore composto WIfI, que vai do 0 ao 9 e pesa todas as variáveis WIfI igualmente. Nesse estudo, 903 pacientes, com 992 membros submetidos a revascularização pela primeira vez, foram divididos em três coortes: (1) todos os pacientes; (2) pacientes submetidos apenas a cirurgia convencional; e (3) pacientes submetidos apenas a um procedimento endovascular. Os novos escores foram os únicos preditores consistentes de mortalidade entre as três coortes, enquanto a classificação WifI da SVS não foi associada à mortalidade em nenhuma das três^14^^,^^26^.

Entretanto, Beropoulis et al.^18^ validaram a capacidade prognóstica dos estágios clínicos de 1 a 4 da classificação WIfI em relação a amputação e mortalidade num subgrupo altamente específico: pacientes não diabéticos tratados por via endovascular. Adicionalmente, Novak et al.^27^ comprovaram que o grau da ferida é o fator mais preditivo da classificação WIfI para a sobrevida do paciente.

### Re-estadiamento após intervenção

Uma validação precoce da classificação WIfI já sugeria sua capacidade para prever os desfechos clínicos após a revascularização^15^. Leithead et al. constataram que, mais do que somente estratificar antes da intervenção, o re-estadiamento com a classificação WIfI da SVS é uma importante ferramenta para prever a perda de membros e avaliar a eficácia da intervenção^28^.

### Eventos de reintervenção, amputação e estenose

Darling et al.^14^^,^^16^, em dois estudos sobre a classificação WIfI, sugeriram que, em pacientes submetidos a revascularização do MI, seja ela aberta ou endovascular, a subida de um grau no estágio clínico da classificação WIfI da SVS mostrava aumento no risco de eventos de reintervenção, amputação e estenose (RAS). Os estudos ainda apoiaram a capacidade do sistema de classificação WIfI da SVS de prever o risco de amputação de 1 ano e as taxas de cicatrização de feridas em pacientes com ICM submetidos a procedimentos de revascularização endovascular infrapoplítea. Outra análise retrospectiva de 302 pacientes não diabéticos submetidos a intervenção vascular entre 2013 e 2014 acrescentou que, nessa subpopulação, a reintervenção ocorreu mais frequentemente no grupo classificado em estágio clínico 3^18^.

É importante frisar que a classificação WIfI da SVS é apenas um dos três componentes necessários à criação de uma sequência de tratamento ao MIA. São necessárias uma estratificação que aborde as comorbidades dos pacientes e outra que avalie o padrão anatômico da doença e sua gravidade^1^^,^^12^. Para tanto, a SVS, a Sociedade Europeia de Cirurgia Vascular e a *World Federation of Vascular Societies* se uniram para criar as *Global Vascular Guidelines*, as novas diretrizes para avaliação e manejo da CLTI, finalizadas e publicadas em 2019^29^. A classificação WIfI, além de ser endossada por tais diretrizes, também será validada em estudos grandes como o *Best Endovascular vs. Best Surgical Therapy in Patients with Critical Limb Ischemia* (BEST- CLI), que já está em reta final de preparação^30^.

## CONCLUSÃO

O sistema de classificação WIfI da SVS continua sendo tema de debate quando o assunto é a melhoria no tratamento aos pacientes portadores de MIA. A maior parte dos estudos de validação da classificação demonstra a associação desse sistema a fatores ligados ao salvamento do membro, como taxas de amputação maior e menor, SLA, predição de eventos RAS e cicatrização de feridas. Esforços conjuntos são ainda necessários para otimizar o tratamento ao paciente com MIA, melhorando a forma como é analisado, estratificado e, consequentemente, manejado clinicamente.
